# Systems Science and Childhood Obesity: A Systematic Review and New Directions

**DOI:** 10.1155/2013/129193

**Published:** 2013-04-23

**Authors:** Asheley Cockrell Skinner, E. Michael Foster

**Affiliations:** ^1^Department of Pediatrics, The University of North Carolina at Chapel Hill, CB 7225, Chapel Hill, NC 27599, USA; ^2^Department of Health Care Organization and Policy, School of Public Health, University of Alabama at Birmingham, Birmingham, AL 35294, USA

## Abstract

As a public health problem, childhood obesity operates at multiple levels, ranging from individual health behaviors to school and community characteristics to public policies. Examining obesity, particularly childhood obesity, from any single perspective is likely to fail, and systems science methods offer a possible solution. We systematically reviewed studies that examined the causes and/or consequences of obesity from a systems science perspective. The 21 included studies addressed four general areas of systems science in obesity: (1) translating interventions to a large scale, (2) the effect of obesity on other health or economic outcomes, (3) the effect of geography on obesity, and (4) the effect of social networks on obesity. In general, little research addresses obesity from a true, integrated systems science perspective, and the available research infrequently focuses on children. This shortcoming limits the ability of that research to inform public policy. However, we believe that the largely incremental approaches used in current systems science lay a foundation for future work and present a model demonstrating the system of childhood obesity. Systems science perspective and related methods are particularly promising in understanding the link between childhood obesity and adult outcomes. Systems models emphasize the evolution of agents and their interactions; such evolution is particularly salient in the context of a developing child.

## 1. Introduction

Childhood obesity is widely considered a critical public health issue, but efforts to address it have yielded few clear-cut answers either for clinical care or public health. Reductions in childhood obesity have been attempted through a variety of means, ranging from clinical interventions to public policies. These failures to some degree reflect a misunderstanding of the nature of obesity itself but also more deeply how the multilevel nature of the phenomenon influences the way research must approach the problem. 

As a public health problem, obesity operates at multiple levels, ranging from individual health (and other) behaviors to parent-child interactions to community and school characteristics to local, state, and federal public policies. These different levels influence each other in ways that are direct and intended as well as through subtle, unanticipated effects that appear over time. Take, for example, efforts to improve access to play spaces to reduce childhood obesity. Building a public park may offer individuals living within walking distance an opportunity to walk even more as well as play structures for children. Disruptions in traffic patterns, however, may make it more difficult for those living somewhat further away to walk at all. Matters may become more complicated if the park stimulates the development of relevant amenities (such as bars and ice cream parlors). Over time, housing prices in the area of the park may improve, changing the socioeconomic and racial composition of the neighborhood. Understanding the effects of the park requires considering both timing and the effect for whom. Even those originally benefiting from the park eventually may drive to the park (increasing traffic) and walk some, but less than they had walked otherwise. And of course, the park has a range of other effects that policy makers and society value other than obesity, such as the time families spend together. Clearly, assessing whether individuals who live near the park exercise in the park a year after it is built is a very limited—indeed even misleading—perspective on its impact and merits. 

Systems science offers a means of identifying and understanding the complex relationships involved in public health policies. It recognizes that policies are based on complex, interdependent, and evolving relationships and include heterogeneous agents (e.g., individuals, companies, or civic associations) acting in their own perceived self-interests. Time matters, as relationships among the agents have a history and, as a result, can develop stability or even inertia. In a complex system, intervention in one aspect will have unanticipated effects, often delayed and nonlinear. Such effects are not exceptions but the norm [1]. Feedbacks play a major role in the systems perspective, and they may be positive or negative. Negative effects often act to restore the system to its prior state and thus thwart any effort to change part of the system. Systems scientists call this tendency “policy resistance” [1, 2]. As with our public park, communities have a life of their own, reflecting and shaping the behavior of individuals within them.

Many of these notions are particularly salient for the study of obesity, especially obesity among children, which combines the complex nature of obesity with the developmental aspects of childhood. Obesity prevention and treatment among children has a long history of disappointing results, and such failures come as no surprise to systems scientists. Much of the research addressing childhood obesity—and thus the dissemination of this work to the practitioners who most need it—is conducted and grounded intellectually in traditional clinical and public health environments. 

This paper describes the past use of systems thinking and models in health and obesity research and lays out an approach for future research, grounded in a systems model resting on nine properties characterizing obesity among children. We first review those properties and systematically review the use of systems tools in obesity research. We then present a systems model of childhood obesity.

## 2. Systems Science Methodologies 

Systems science represents a comprehensive perspective for understanding broad social and health problems. One key tool is simulation modeling, grouped into three broad conceptual paradigms: system dynamics modeling (SDM), agent-based modeling (ABM), and discrete event simulation (DES) [3]. Many problems can be framed so that any of the three methods could be used, but the methods have distinguishing features that lend themselves to certain types of problems. 

SDM is the oldest and arguably most common of these three methods [4]. Models in SDM emphasize causal feedback loops and unintended consequences. SDM acknowledges the exchange of resources among agents to produce both desirable and undesirable outcomes. This method is distinguished by an emphasis on stocks and flows. Stocks represent accumulations and characterize a system at a point in time; they provide systems with inertia and memory [2].

DES also describes complex systems over time and the conversion of inputs into outputs. DES revolves around “events” [5] that involve entities (e.g., individuals, firms) moving between different states (e.g., health, production). Key features are that different stages or different entities involve shared resources and the importance of timing. Entities move along different stages in the process in sequence, exiting one stage and moving to the next when space is available. 

Agent-based modeling focuses on the broadly characterized pattern of interactions among individuals [6]. These models emphasize the “influence” individuals exert on each other, whether communication of diseases or interest in purchasing certain goods. These models can illuminate patterns of aggregate behavior that emerge from simple models of individual behavior; some of the former may be relatively robust to alternative models of the latter. This approach is dynamic: individuals or a population may accumulate experience that shapes further choices and development. 

The three broad paradigms have many common elements, such as understanding unanticipated consequences of choices or aggregate patterns of behavior that were not explicable when judged from the ground up (at the level of the individual agent or entity). All have advantages over alternative approaches, such as Markov models one finds in economic evaluation of health policies [7]. The three paradigms have remained somewhat distinct partly for conceptual reasons, but these differences are smaller than in the underlying programming approaches. Current programming allows the various types of systems science to be combined, creating even more powerful ways of examining policies.

## 3. Systems Science in Health

 Systems science methodologies are increasingly used in health services and public health research. This growth has been stimulated by a recent joint report from the Institute of Medicine and the National Academy of Engineering, “Building a Better Delivery System” [8]. Health care and public health have increasingly been recognized as complex systems, where addressing problems requires considering the entire system. 

Some studies have demonstrated the utility of systems science models in a variety of health and health care situations. These include influence of individuals' interactions on risky drinking behavior [9], interventions to reduce childhood caries [10], and how altering personnel affects emergency department throughput [11]. The breadth of potential use for systems science models in health care demonstrates how they may become a critical tool in the development of health policy, including childhood obesity policy [12]. 

### 3.1. Nine Properties on Which to Build a Systems model of Obesity

Obesity in general and childhood obesity in particular raise important issues of interest to systems science [13–15]. Conventional obesity research suffers from many of the limitations that affect any effort to understand systems without a system perspective. At its essence, obesity raises key systems questions for several reasons. A report from the Institute of Medicine, “Bridging the Evidence Gap in Obesity Prevention”, discusses the systems science perspective and the needs in obesity research [16]. Although not directly drawn from “Bridging the Evidence Gap”, we identify and propose the following nine properties as critical to obesity. Importantly, the effect of obesity-related public health policies requires considering all of the properties.

1. Obesity prevention and treatment is a common resource allocation problem, and a full understanding of the entire system is required to make appropriate allocations.
2. Obesity both shapes and reflects a range of other issues. That is, it is both an outcome of certain conditions and a cause of others.
3. Both obesity and its consequences are evolving developmental processes, which offer multiple points for interventions.
4. Obesity is determined in a social process that involves families and peers.
5. Obesity occurs in an environment that moderates the influence of processes operating at other levels.
6. Many interventions can be assessed only over time and have unanticipated effects.
7. People are highly heterogeneous in their biological systems and predispositions toward obesity.
8. Individuals all have a defined “space” within the system, both among other individuals and geographically, and that place influences obesity.
9. Individuals have imperfect knowledge about obesity.

The properties were not identified because of their link to systems science, but rather because they are fundamental aspects of obesity. In their discussion of systems science in public health, Luke and Stamatakis [17] present some key features of systems science models. These items are listed in Table 1 and mapped to the above obesity properties. Foremost, obesity reflects a nexus of forces that all act to have individuals consume more energy than they expend. These forces include individual, family, and community. That obesity reflects a multilevel process is well established. What is lacking is an analytical method for understanding this process. Systems science is a natural fit for obesity research.

### 3.2. Past Use of Systems Tools in Obesity Research

Levy and colleagues [18] recently reviewed simulation models in obesity research, demonstrating a wide variety of model types used to address obesity. Our goal is to build upon this knowledge to provide a systematic examination of how systems science methods have been used to examine obesity from a clinical and public health perspective.

## 4. Methods

We performed a systematic literature review of studies that used systems science methodologies to study obesity in the context of public health. Although few studies use a full systems science perspective, we attempted to identify studies that are developing the pieces of useful models.

### 4.1. Search Strategy

We chose to focus on the types of models used in systems science as the basis for our search strategy. We searched PubMed and Web of Science (ISI) through March 2012 using “obesity” AND the following key words and phrases: (“simulation model”), (“agent-based”), (“discrete event”), (“system dynamics” OR “systems dynamics”), (“network analysis”), (“Markov simulation”), (“dynamic microsimulation”), and (“systems science”). For our purposes here, we chose not to search other databases in fields such as economics. Although these areas may include additional studies presenting relevant models, we hope to capture findings that are most consistently accessed by the audience of public health and clinical scientists seeking to understand—and intervene in—obesity.

Although network analysis and Markov simulation are not, themselves, systems science methods, we have included them in the keywords because studies using these methods may approach obesity from one of the nine properties listed in Table 1. Network analysis, in particular, provides critical information about the relationships among agents in the system. We also recognize that this strategy may not represent comprehensive coverage of all obesity-related “systems science” studies. However, we believe it provides a reasonable representation of systems science as currently used in public health obesity research.

### 4.2. Inclusion and Exclusion Criteria

Inclusion of articles was based on the use of techniques that addressed any of the properties of systems science listed in Table 1. Our goal is to identify research that approaches obesity from a systems perspective, even incrementally. In order to be included in our review, studies had to meet all of the following criteria:

1. must examine obesity in the framework of systems science or using one or more of the properties of systems science as described in Table 1,
2. include original analyses, rather than discussing only how systems science could be used, and
3. must include obesity in the model, as a predictor and/or outcome. Although obesity-related behaviors can be (and often are) modeled without obesity included, our intention is to see and determine how models have used obesity specifically.

We excluded studies that examined only the biological system of the individual. Although systems science approaches can be and have been used to understand the physiological mechanisms of obesity, we are primarily interested here in a discussion of clinical and public health, and of understanding the macrolevel use of systems science. We also excluded studies not published in English.

### 4.3. Review Process

One author (ACS) reviewed the abstracts of all articles that were retrieved from the search results to determine if they met inclusion criteria. We then obtained the complete article and applied the exclusion criteria to create the final list of included articles. We then reviewed the references of our included studies to identify additional articles of interest. 

## 5. Results

**Figure 1:**
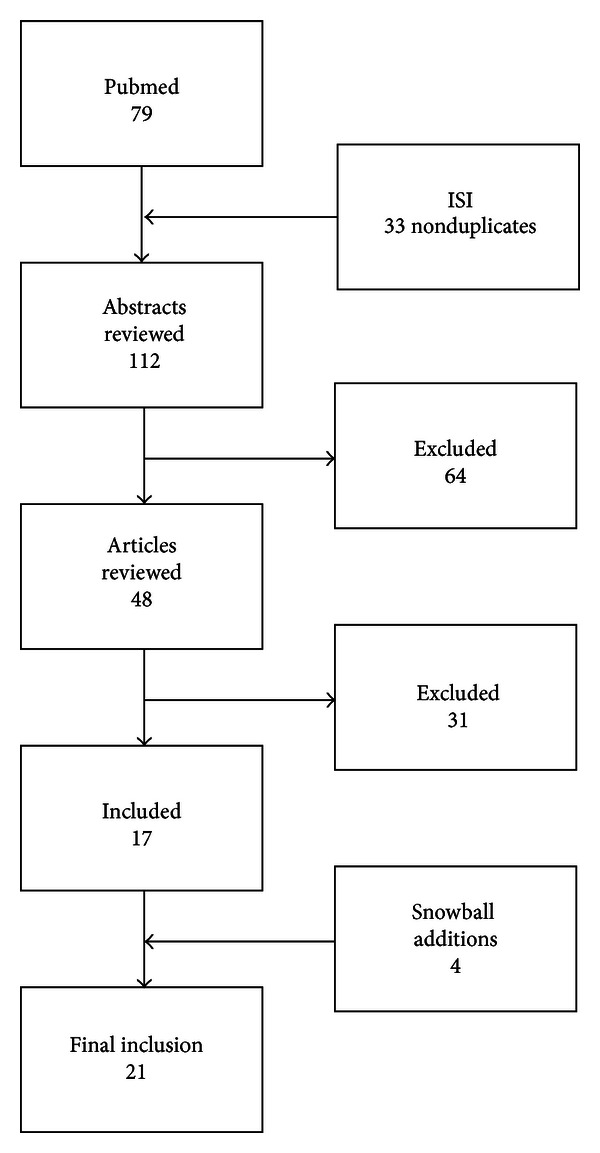
Schematic of search process.

We identified 112 articles using the search criteria described above (Figure 1). We excluded 64 after abstract review: the most common reasons for exclusion were that the article addressed physiology and did not address obesity or were commentaries about systems science models. Of the 48 remaining articles, we excluded an additional 31 articles, primarily because they did not include obesity in their analyses, leaving 17 articles included in our review. We reviewed the references in the 17 articles to identify any potential articles that may have been missed using our other search methods, resulting in four additional articles included in our review.

The 21 included studies addressed four general areas of systems science in obesity: (1) translating interventions to a large scale, (2) the effect of obesity on other health or economic outcomes, (3) effect of geography on obesity, and (4) the effect of social networks on obesity.  Table 2 demonstrates the properties of systems science that each article addressed.

### 5.1. Translating Obesity Interventions to a Large Scale

The most common systems science studies examine how interventions could change obesity or obesity-related outcomes on a large scale. Overall, efforts to use true systems science approaches have been most common in this area. These studies fall into two general categories: (a) those examining the effect of a hypothetical intervention or change and (b) those aggregating results of previously studied interventions to a large scale.

#### 5.1.1. Hypothetical Interventions

Goldman and colleagues used a dynamic microsimulation model to examine the effect of risk factor prevention in Americans aged 51 and older [19]. The model defined an individual's probability of transitioning from one state (e.g., health) to another (e.g., cardiovascular disease) and demonstrated reductions in disease burden and costs from hypothetical treatment scenarios. 

Goris and colleagues focused on the interaction of multiple levels in their examination of the effect of television food advertising on obesity in children in six countries [20]. By using estimated obesity prevalence and differences in advertising, they modeled the proportion of obesity related to advertising, demonstrating significant reductions with the hypothetical elimination of television food advertising. Veerman and colleagues use a similar framework to examine the effects of reducing television food advertising on obesity in US children. [21] They find that reducing television advertising to zero would result in a reduction of the prevalence of obesity in children by 2.5 to 6.5 percentage points. 

Jones and colleagues describe the development of dynamic simulation model of population-level diabetes development and control [22]. This well-defined model estimates future increases in diabetes prevalence and diabetes complications. Additionally, the model is used to test the effect of several hypothetical scenarios of improvements in diagnosis, management, and reductions in obesity, all of which reduce the prevalence of diabetes and/or diabetes complications. 

#### 5.1.2. Previously Studied Interventions

Bemelmans and colleagues developed a dynamic simulation model to examine the effects and cost-effectiveness of applying previously developed intervention on a national level for obesity in The Netherlands [23]. Using states based on age, weight, and physical activity, they simulated how population-level interventions would affect the transition between states and the resulting effects on health outcomes and costs. 

Hoerger and colleagues developed a Markov simulation model to examine the progression of diabetes and the effect of diabetes screening in the US population [24]. This dynamic model used several “modules”—screening, prediabetes, and diagnosed diabetes—and different, previously studied interventions for each state. They were able to demonstrate the cost-effectiveness of screening and a prevention-focused lifestyle intervention.

Using a similar model to the one described above, Hoerger and colleagues incorporated bariatric surgery in order to examine the effect of this intervention in individuals with newly diagnosed or established diabetes [25]. They find that bariatric surgery appears to be relatively cost-effective for severely obese patients with diabetes although improvements in diabetes decline over time.

Hall and colleagues used a dynamic simulation model to examine a variety of weight-related outcomes and other factors. Specific to public health, they demonstrate that their dynamic simulation (as opposed to linear estimation) shows that a tax on sugar-sweetened beverages would have a much smaller effect on population-level weight than previous reports [26].

### 5.2. Effect of Obesity on Comorbidities/Other Outcomes

Studies to examine the effects of obesity on other outcomes, including comorbidities, have primarily used dynamic simulation models. However, these have not included the feedback loops or complex structures that have been used in studies that aimed to predict the effects of interventions. Rather, they have focused on the probability of changing states (e.g., no disease to disease) and the results on prevalence of conditions or costs.

Fesinmeyer and colleagues used a microsimulation model to examine the contribution of obesity to prostate cancer mortality [27]. Their model is based on previous work showing the relationship between prostate cancer and obesity. They were able to estimate how obesity increases the risk of prostate cancer and that despite the overall decreases in prostate cancer mortality, the declines were limited by the increase in obesity. 

Kong and colleagues developed a disease simulation model of the how obesity affect esophageal cancer [28]. Using previously-studied relationships between obesity and esophageal cancer, they compared the expected trend given constant obesity since 1970 and the observed trend with the increase in obesity. The results showed that about 7% of the cancer cases were attributable to obesity.

Losina and colleagues used a policy model to examine the relationship between knee osteoarthritis, obesity, and morbidity [29]. The model is based on transitions between health states based on the combinations of obesity and arthritis. They showed that both of these conditions, and the combination of the two, had a significant impact on morbidity. 

Neovius and colleagues developed a Markov simulation model to examine premature mortality attributable to obesity and smoking in Swedish men [30]. This simulation of cohort over 40 years demonstrated that a reduction in obesity would yield a reduction in premature deaths, but the reduction was small compared to that of eliminating smoking.

van Baal and colleagues used a chronic disease model to examine the effect of obesity on total lifetime health care costs [31]. Their model allowed comparison of lifetime costs under different scenarios of changes in incidence, health care costs, and relative risks. Although a substantial portion of health care costs can be attributed to obesity, the increased life expectancy with obesity reduction yielded no reduction in lifetime health care costs.

Wang and colleagues used a simulation model based on expected obesity trends in the US and the UK in order to examine the effect of obesity on health and costs [32]. They demonstrate that obesity makes significant contributions to morbidity, mortality, and costs. 

Similarly, Lakdawalla and colleagues used a simulation model to examine life expectancy, disability, and costs associated with obesity at age 70, based on transitions among various disease states [33]. Not surprisingly, the results demonstrate increased costs and fewer disability-free years among obese individuals.

Bibbins-Domingo and colleagues use the Coronary Heart Disease Policy Model to examine how current adolescent obesity will affect future CHD prevalence [34]. They demonstrate varying increases in the prevalence of CHD in adulthood based on different assumptions about obesity change. They also show decreases in CHD prevalence based on successful treatment of hypertension and dyslipidemia.

Thompson and colleagues developed a dynamic model to examine the effect of age and obesity on the risk of developing several obesity-related diseases and the subsequent costs [35]. Their results demonstrated a combined effect of age and obesity on disease risk and projected costs.

### 5.3. Effect of Geography on Obesity

Two studies examined the influence of spatial position on obesity. Edwards and colleagues developed a spatial microsimulation model called SimObesity to examine small-area influences on obesogenic behaviors in the United Kingdom [36]. Their results demonstrate clear differences in how low social capital, obesogenic behaviors, poverty, and deprivation and safety affect obesity in different small areas.

MacDonald and colleagues used network analysis to examine the effect of distance to food outlets on BMI in an urban area of the United Kingdom [37]. They demonstrated very few relationships between distance to a food outlet and BMI.

### 5.4. Effect of Networks on Obesity

Two studies examined the interactions between agents (individuals) in the development of obesity. Christakis and colleagues developed a social network analysis to examine the spread of obesity among individuals in the Framingham Heart Study [38]. They demonstrated clear ties in the development of obesity among individuals with social relationships, particularly for same-sex friends, spouses, and siblings. Their model included geographic distance, which indicates that the immediate environment was less important than the social environment. 

Valente and colleagues extended these findings to examine obesity among adolescents and their friends [39]. Although they did not have the benefit of longitudinal data, they were able to demonstrate significant clustering, with adolescents who have friends who are overweight more likely to be overweight themselves.

## 6. Discussion

A limited amount of research addresses obesity from a systems science perspective. Conspicuously few studies examine childhood obesity from a systems perspective, with only two focusing on children—one examining a hypothetical intervention regarding food advertising and the other showing network clustering of obesity in adolescents. System dynamics modeling is the most common, consistent with its longer history of use in research. However, the largely incremental approaches used in current systems science lay a foundation for future work, and an examination of the shortcomings of current research provides critical insight into how such approaches can be used in ways that yield maximum benefit.

A myopic, ground-level view of obesity leads to interventions likely to fail; worse still, that same perspective infuses research, making it difficult for researchers to develop a system-level perspective. A program seems to fail; yet that intervention may very well have been necessary but not sufficient to change the behaviors that influence energy intake and obesity as a result.

What does a system-level perspective reveal about obesity? Obesity in an individual does not occur in isolation. That individual acts within a particular genetic, social, and environmental milieu. Although understanding isolated factors is useful, how we improve obesity from a public health perspective requires a much deeper examination of how all these factors interact. 

One of our inclusion criteria—that obesity be either an outcome or a predictor—reveals a critical failure in our current understanding of the obesity epidemic. The world for which obesity interventions are designed focuses heavily on obesity as an outcome. However, in the real world—the system in which people live—obesity is both a predictor and an outcome. It is this feedback loop that is critical to understanding how to address obesity.

Another critical failure in understanding how to address the childhood obesity epidemic is the lack of long-term studies that demonstrate the effect of interventions in childhood on adult obesity and disease. This is, of course, due to the difficulty in developing long-term studies, and exploring the effect of the entire menu of childhood interventions on adult health is likely an impossible task.

### 6.1. A Model of Childhood Obesity for Systems Science

**Figure 2:**
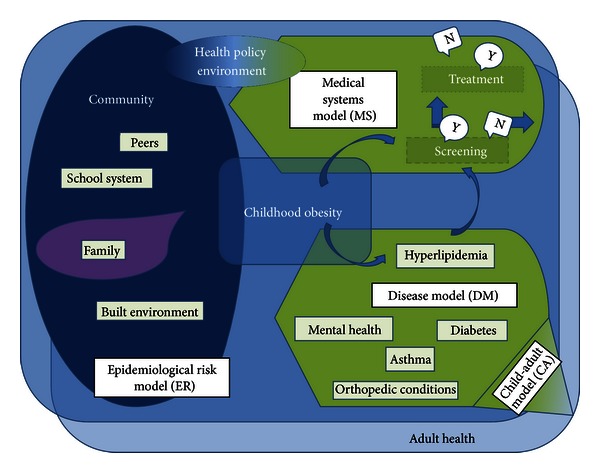
Systems model of childhood obesity.

Overall, systems science is developing a clear foundation for application in obesity. Using systems science methods is most critical in childhood obesity, where the effects of behaviors or interventions on long-term outcomes cannot be fully tested using standard research methods. the development of effective interventions requires an understanding of the physical and social environments, the role of the medical system, the progression of disease, and the effect of this system in childhood on future adult outcomes. In order to guide future systems science approaches, we have developed a model that includes the overall system in which childhood obesity develops and perpetuates (Figure 2).

Figure 2 describes the conceptual model on which systems science models of childhood obesity can build. It is built upon the nine properties of obesity research discussed earlier, as well as the limitations identified in the literature review. The model as a whole (1) considers the effect of allocating resources to different areas for prevention and treatment, (2) demonstrates that obesity is both a cause and an outcome, and (3) that obesity is a dynamic, developmental process; the (4) relationships among individuals and (5) their interactions with the environment can affect the components other than behavior and obesity itself. By combining multiple submodels, it (6) emphasizes the complex relational structures and (7) the heterogeneity of the individual in their risk for obesity and for related diseases. Finally, (8) each individual has a “space”—both geographically and among others, and (9) the knowledge available to the individual can influence all other aspects of the system.

### 6.2. A Vision for Future Research

Limitations of current research are the basis for development of our vision of future research, which is reflected in our model of childhood obesity. Specifically, current studies, for the most part, do not combine the effects of interventions with the resulting outcomes. Systems science methodologies can bring to public health and obesity the ability to model how a particular intervention might affect a larger population—including interaction with other interventions, implementation, how individuals interact with others, and what health improvements and cost differences would be attributable to that intervention. A second limitation with the current approaches is that there is little consistency in the outcomes predicted in terms of time, such as health expenditures. These models yield vastly different interpretations, from obesity being a significant contributor to health care costs to no differences in lifetime expenditures. A third limitation is that current studies examining what influences behaviors have typically not extended the results to the effect on obesity. Although such studies [40–46] provide important foundations to understanding how environments and networks affect behaviors, using these results in public health requires additional understanding of how changes in behaviors result in obesity changes. The limitations of the current research should only be construed in terms of their limitations toward a broad understanding of childhood obesity. Understanding of systems requires research on the individual components as well as the broader picture. Current research is not inherently limited—it simply has not been used to its maximum potential. Systems science is one way to expand current knowledge in ways highly applicable to policy development.

Our description of a hypothetical park hardly strains the imagination of anyone who has been to a park. Indeed, our suspicion (and hope) is that the reader thought of examples of places s/he had been as s/he read that paragraph. As noted, however, a single study or line of research generally does not capture the complexity implied by our example. To do so requires a new vision for obesity research that we outline here.

First and foremost, no single study can provide the data needed to understand these processes. It has become clear that in many areas, research has to be combined in an overarching vision. A single study has to offer a partial view for a range of reasons (including but not limited to research budgets). In terms of childhood obesity, which results from actions at multiple levels, appropriately combining multiple studies is the only way to fully understand the problem. Related to this, a vision that combines multiple studies offers a means to prioritize research. When one tries to complete such a model, it is often quite clear that a duplicative multitude of studies are available for one part of a model with little, if any, research on another part. 

Second, no single field can provide the theoretical insights, methods, and guidance required for this vision of research. Studies outside of the “obesity literature” have an important role to play in the model. In the case of our park, it seems clear that expertise in a range of topics is needed–exercise physiologists, nutritionists, recreation scientists, transportation researchers, real estate experts, to name just a few. 

Third, research in this area needs a broader understanding and greater emphasis on time. Research at a microlevel points the way in that regard. A key issue with weight loss is not losing weight but maintaining the loss. A dynamic model (biological and behavioral) highlights the role of feedback to understand how and why individuals tend to regain weight lost [47]. Research that begins with the presumption that "all else is held constant" is doomed to fail. Systems are continuously changing. Obesity well illustrates how quickly population health can change. For example, the percentage of children who are obese in the United States increased by nearly one-third between 1999-2000 and 2007-2008. In the last two years, however, rates have actually declined [48]. Obesity itself is an ever-moving, dynamic target which means that research based on ceteris paribus will never reach maximum impact—and obesity is but a single summary of a large, complex system.

This work also rests on a broader understanding of the development of research as well. A model like we are describing here would not be developed, used to answer a specific question or test a single hypothesis, and then put aside. Rather it would continue to grow as new studies shed (better) light on key aspects of it. Indeed new agents or participants would enter the model over time. Currently, we often think of the research process as a series of studies, rather than one study that both continues perpetually (not unlike a longitudinal cohort study) and changes constantly (very much unlike most longitudinal studies). Pushing forward such as innovative and forward-thinking research will require understanding of the nature of systems science by the overall research community.

One might argue that the vision of research here is too ambitious—that it involves the determinants and consequences of obesity for everyone at different time points in the lives of individuals and communities. However, this vision is exactly what we want public health policy makers to do—to make decisions that improve the public health now and in the future. There are many examples where a failure to embrace such a vision leaves key questions unanswered. For example, translational research involves developing clinical interventions and implementing them in public health systems. Often such efforts to make this transition fail and we know relatively little about how to make successful transitions from bench to bedside (type 1) and bedside to curbside (type 2). With a systems model and the understanding it provides, these failures, if not prevented, then could inform the systems model and improve the chances of future transitions.

## 7. Conclusion

What does a system-level perspective reveal about childhood obesity? Obesity in an individual does not occur in isolation. That individual acts within a particular genetic, social, and environmental milieu. Although understanding isolated factors is useful, how we improve obesity from a public health perspective requires a much deeper examination of how all these factors interact. Systems science methods have been used to help understand the complex physiology of obesity within an individual [49, 50]. Ideally, we would eventually combine these as additional levels to the overall “obesity system.” However, to do so will require significant interdisciplinary teamwork, including basic scientists, clinical scientists, public health researchers, and researchers with systems science knowledge and the skills to apply it to many different levels, and only then will we improve our ability to undertake such examinations, from a truly dynamic, system perspective. 

## Figures and Tables

**Table 1:** Systems science properties and nine properties of obesity.

Systems science properties	Obesity properties
Breadth	Obesity prevention and treatment should be considered a common resource allocation problem
Feedback Loops	Obesity shapes and reflects a range of other issues
Dynamic systems in real time	Obesity and its consequences are developmental processes
Interactions of individuals actors	Obesity is a social process involving families, peers, and other individuals
Interactions between multiple levels	Obesity operates within a community environment that moderates family and individual levels
Complex relational structures	Interventions can only be assessed over time and can have unanticipated results
Heterogeneous actors	People are heterogeneous in their biological and behavioral predispositions towards obesity
Spatial	Individuals all have a space within the system
Bounded rationality	Individuals have imperfect knowledge about obesity

**Table 2:** Properties of systems science addressed in the included studies.

	Breadth	Loops	Dynamic systems	Interaction of agents	Multiple levels	Complex structures	Heterogeneous actors	Spatial	Bounded rationality
Interventions									
Goldman et al.			X						
Goris et al.					X				
Veerman et al.					X				
Jones et al.	X	X	X			X			
Bemelmans et al.			X		X	X			
Hoerger et al.	X	X	X			X			
Hoerger et al.	X	X	X			X			
Hall et al.			X						
Comorbidities/Costs									
Fesinmeyer et al.			X						
Kong et al.			X						
Losina et al.			X						
Neovius et al.			X						
Van Baal et al.			X						
Wang et al.			X						
Bibbins-Domingo et al.			X						
Lakdawalla et al.			X						
Thompson et al.			X						
Geography									
Edwards and Clarke								X	
MacDonald et al.								X	
Networks									
Christakis and Fowler				X				X	
Valente et al.				X				X	

## References

[1] Sterman JD (2006). Learning from evidence in a complex world. *American Journal of Public Health*.

[2] Sterman J (2000). *Business Dynamics: Systems Thinking and Modeling for a Complex World*.

[3] Borshchev A, Filippov A From system dynamics and discrete event to practical agent based modeling: reasons, techniques, tools.

[4] Forrester JW (1958). Industrial dynamics: a major breakthrough for decision makers. *Harvard Business Review*.

[5] Banks J, Carson JS (1984). *Discrete-Event System Simulation*.

[6] Epstein JM (1999). Agent-based computational models and generative social science. *Complexity*.

[7] Barton P, Bryan S, Robinson S (2004). Modelling in the economic evaluation of health care: selecting the appropriate approach. *Journal of Health Services Research and Policy*.

[8] National Academy of Engineering (2005). *Building a Better Delivery System: A New Engineering/Health Care Partnership*.

[9] Gorman DM, Mezic J, Mezic I, Gruenewald PJ (2006). Agent-based modeling of drinking behavior: a preliminary model and potential applications to theory and practice. *American Journal of Public Health*.

[10] Hirsch GB, Edelstein BL, Frosh M, Anselmo T (2012). A simulation model for designing effective interventions in early childhood caries. *Preventing Chronic Disease*.

[11] Day TE, Al-Roubaie AR, Goldlust EJ (2013). Decreased length of stay after addition of healthcare provider in emergency department triage: a comparison between computer-simulated and real-world interventions. *Emergency Medicine Journal*.

[12] Maglio PP, Mabry PL (2011). Agent-based models and systems science approaches to public health. *American Journal of Preventive Medicine*.

[13] Gortmaker SL, Swinburn BA, Levy D (2011). Changing the future of obesity: science, policy, and action. *The Lancet*.

[14] Hammond RA (2009). Complex systems modeling for obesity research. *Preventing Chronic Disease*.

[15] Huang TT, Drewnosksi A, Kumanyika S, Glass TA (2009). A systems-oriented multilevel framework for addressing obesity in the 21st century. *Preventing Chronic Disease*.

[16] Kumanyika SK, Parker L, Sim LJ (2011). *Bridging the Evidence Gap in Obesity Prevention: A Framework to Inform Decision Making*.

[17] Luke DA, Stamatakis KA (2012). Systems science methods in public health: dynamics. *Annual Review of Public Health*.

[18] Levy DT, Mabry PL, Wang YC (2011). Simulation models of obesity: a review of the literature and implications for research and policy. *Obesity Reviews*.

[19] Goldman DP, Zheng YH, Girosi F (2009). The benefits of risk factor prevention in Americans aged 51 years and older. *American Journal of Public Health*.

[20] Goris JM, Petersen S, Stamatakis E, Veerman JL (2010). Television food advertising and the prevalence of childhood overweight and obesity: a multicountry comparison. *Public Health Nutrition*.

[21] Veerman JL, van Beeck EF, Barendregt JJ, MacKenbach JP (2009). By how much would limiting TV food advertising reduce childhood obesity?. *European Journal of Public Health*.

[22] Jones AP, Homer JB, Murphy DL (2006). Understanding diabetes population dynamics through simulation modeling and experimentation. *American Journal of Public Health*.

[23] Bemelmans W, van Baal P, Wendel-Vos W (2008). The costs, effects and cost-effectiveness of counteracting overweight on a population level. A scientific base for policy targets for the Dutch national plan for action. *Preventive Medicine*.

[24] Hoerger TJ, Hicks KA, Sorensen SW (2007). Cost-effectiveness of screening for pre-diabetes among overweight and obese U.S. adults. *Diabetes Care*.

[25] Hoerger TJ, Zhang P, Segel JE, Kahn HS, Barker LE, Couper S (2010). Cost-effectiveness of bariatric surgery for severely obese adults with diabetes. *Diabetes Care*.

[26] Hall KD, Sacks G, Chandramohan D (2011). Quantification of the effect of energy imbalance on bodyweight. *The Lancet*.

[27] Fesinmeyer MD, Gulati R, Zeliadt S, Weiss N, Kristal AR, Etzioni R (2009). Effect of population trends in body mass index on prostate cancer incidence and mortality in the United States. *Cancer Epidemiology Biomarkers and Prevention*.

[28] Kong CY, Nattinger KJ, Hayeck TJ (2011). The impact of obesity on the rise in esophageal adenocarcinoma incidence: estimates from a disease simulation model. *Cancer Epidemiology Biomarkers & Prevention*.

[29] Losina E, Walensky RP, Reichmann WM (2011). Impact of obesity and knee osteoarthritis on morbidity and mortality in older Americans. *Annals of Internal Medicine*.

[30] Neovius K, Rasmussen F, Sundström J, Neovius M (2010). Forecast of future premature mortality as a result of trends in obesity and smoking: nationwide cohort simulation study. *European Journal of Epidemiology*.

[31] van Baal PHM, Polder JJ, de Wit GA (2008). Lifetime medical costs of obesity: prevention no cure for increasing health expenditure. *PLoS Medicine*.

[32] Wang YC, McPherson K, Marsh T, Gortmaker SL, Brown M (2011). Health and economic burden of the projected obesity trends in the USA and the UK. *The Lancet*.

[33] Lakdawalla DN, Goldman DP, Shang B (2005). The health and cost consequences of obesity among the future elderly. *Health Affairs*.

[34] Bibbins-Domingo K, Coxson P, Pletcher MJ, Lightwood J, Goldman L (2007). Adolescent overweight and future adult coronary heart disease. *The New England Journal of Medicine*.

[35] Thompson D, Edelsberg J, Colditz GA, Bird AP, Oster G (1999). Lifetime health and economic consequences of obesity. *Archives of Internal Medicine*.

[36] Edwards KL, Clarke GP (2009). The design and validation of a spatial microsimulation model of obesogenic environments for children in Leeds, UK: simObesity. *Social Science and Medicine*.

[37] MacDonald L, Ellaway A, Ball K, MacIntyre S (2011). Is proximity to a food retail store associated with diet and BMI in Glasgow, Scotland?. *BMC Public Health*.

[38] Christakis NA, Fowler JH (2007). The spread of obesity in a large social network over 32 years. *The New England Journal of Medicine*.

[39] Valente TW, Fujimoto K, Chou CP, Spruijt-Metz D (2009). Adolescent affiliations and adiposity: a social network analysis of friendships and obesity. *Journal of Adolescent Health*.

[40] Miyake KK, Maroko AR, Grady KL, Maantay JA, Arno PS (2010). Not just a walk in the park: methodological improvements for determining environmental justice implications of park access in New York City for the promotion of physical activity. *Cities and the Environment*.

[41] Auchincloss AH, Riolo RL, Brown DG, Cook J, Roux AVD (2011). An agent-based model of income inequalities in diet in the context of residential segregation. *American Journal of Preventive Medicine*.

[42] Larsen K, Gilliland J (2008). Mapping the evolution of “food deserts” in a Canadian city: supermarket accessibility in London, Ontario, 1961–2005. *International Journal of Health Geographics*.

[43] Maroko AR, Maantay JA, Sohler NL, Grady KL, Arno PS (2009). The complexities of measuring access to parks and physical activity sites in New York City: a quantitative and qualitative approach. *International Journal of Health Geographics*.

[44] Sadler RC, Gilliland JA, Arku G (2011). An application of the edge effect in measuring accessibility to multiple food retailer types in Southwestern Ontario, Canada. *International Journal of Health Geographics*.

[45] Yang Y, Roux AVD, Auchincloss AH, Rodriguez DA, Brown DG (2011). A spatial agent-based model for the simulation of adults' daily walking within a city. *American Journal of Preventive Medicine*.

[46] Zenk SN, Schulz AJ, Matthews SA (2011). Activity space environment and dietary and physical activity behaviors: a pilot study. *Health & Place*.

[47] Abdel-Hamid TK (2009). *Thinking in Circles about Obesity: Applying Systems Thinking to Weight Management*.

[48] Ogden CL, Carroll MD, Kit BK, Flegal KM (2012). Prevalence of obesity and trends in body mass index among US children and adolescents, 1999–2010. *Journal of the American Medical Association*.

[49] Abdel-Hamid TK (2002). Modeling the dynamics of human energy regulation and its implications for obesity treatment. *System Dynamics Review*.

[50] Abdel-Hamid TK (2003). Exercise and diet in obesity treatment: an integrative system dynamics perspective. *Medicine and Science in Sports and Exercise*.

